# Prediction of Tubal Rupture in Ectopic Pregnancy Using Methotrexate Treatment Protocols and Hematological Markers

**DOI:** 10.3390/jcm12206459

**Published:** 2023-10-11

**Authors:** Sevtap Seyfettinoglu, Fikriye Işıl Adıguzel

**Affiliations:** 1 Department of Gynecologic Oncology, University of Health Science, Adana City Training and Research Hospital, 01230 Adana, Turkey; 2 Department of Obstetrics and Gynecology, University of Health Science, Adana City Training and Research Hospital, 01230 Adana, Turkey; f.adiguzel1@saglik.gov.tr

**Keywords:** ectopic pregnancy, methotrexate, neutrophil-to-lymphocyte ratio, tubal rupture

## Abstract

Ectopic pregnancy is a pregnancy complication in which the embryo implants outside the uterine cavity. Although medical treatment is chosen first, sometimes a rupture may occur, and surgical treatment may be required. The parameters to predict rupture have been the subject of many studies. This study aimed to compare the efficacy of different methotrexate protocols in the treatment of ectopic pregnancy and determine the parameters and methotrexate treatment protocols that can predict the risk of rupture. A total of 128 patients diagnosed with ectopic pregnancy were included in this study. Patients were separated into three categories based on their treatment protocols. Regarding the occurrence of rupture, all three groups were compared. The hematological parameters and methotrexate treatment protocols were analyzed and compared between groups. The mean age was 31.9 years. Parity was significantly higher in patients who received multiple doses of methotrexate compared to the other groups. There were significant variations observed among the groups regarding parity, initial β-hCG values, hematocrit (HTC), and mean corpuscular volume (MCV) (*p* = 0.048, *p* < 0.001, *p* = 0.019, and *p* = 0.047, respectively). According to receiver operating characteristic analysis, neutrophil-to-lymphocyte ratio (NLR) levels were significantly associated with histopathologically confirmed tubal rupture (*p* < 0.05). NLR levels should be examined in ectopic pregnancy, and the possibility of rupture should be considered in cases with high NLR levels. The potential of NLR to predict ectopic pregnancy rupture should be explored in multicenter prospective studies.

## 1. Introduction

Ectopic pregnancy (EP) is a common acute abdominal disorder in obstetrics and gynecology, with an incidence of approximately 2%. It is a common cause of death, accounting for 4–10% of maternal deaths [1]. Therefore, making a diagnosis and treatment plan is very important [2]. The standard approach to diagnosis includes ultrasound imaging and monitoring β-human chorionic gonadotropin (β-hCG) levels. Early diagnosis can help reduce maternal mortality rates. Many laboratory parameters and biomarkers have been investigated for more effective diagnoses. However, a clear predictor that can be used in place of β-hCG has not been obtained [3,4,5,6]. In addition to biomarkers, endometrial sampling can be used to diagnose EP and allows for the exclusion of intrauterine pregnancy [7]. Usually, β-hCG measurements are combined with US when diagnosing EP. If there is no evidence for intrauterine pregnancy and β-hCG levels are >2000 mIU/mL, ectopic pregnancy is a highly probable diagnosis. In 95% of cases, the EP implants within the tubal lumen [2,8]. Complete blood count (CBC) parameters can be used to diagnose EP. For example, white blood cell count is higher in ectopic pregnancies. Similarly, platelet distribution width (PDW) has also been studied as a marker for ectopic pregnancy [8,9]. However, there are not enough data to use these parameters for diagnosis.

There are three treatment approaches for ectopic pregnancy: expectant management, medical treatment, and surgery. Methotrexate (MTX), used in the medical treatment of EP and considered the gold standard, is an antitumor drug with a molecular structure similar to folic acid [5]. The antitumor mechanism of MTX involves reversible competitive action on the Dihydrofolate Reductase enzyme, leading to the impairment of purine and pyrimidine rings synthesis and resulting in the inhibition of cell division [7]. Three MTX protocols (single, double, and multiple doses) are generally accepted for treating ectopic pregnancies [10]. The number of doses administered may vary depending on the patient’s β-hCG value. Patients with high β-hCG levels may benefit from a double dose of MTX therapy. Multi-dose regimens vary in dosing and include co-administration with leucovorin (folinic acid). Leucovorin reduces the negative effects of MTX but, at the same time, reduces the effectiveness of treatment [11,12]. A single-dose protocol is preferred over multiple-dose and two-dose protocols, as it does not require leucovorin administration and more clinic visits [5]. In a single-dose protocol, 50 mg/m^2^ of MTX is administered intramuscularly. Following administration, β-hCG levels are monitored. If β-hCG levels, fetal cardiac activity, presence of free fluid, and an EP diameter greater than 3.5 cm are not reduced, treatment is considered to have failed [13,14]. In patients who receive a single dose of MTX, β-hCG measurement is repeated on the 4th and 7th days of treatment, and a second dose is administered to patients with a decrease of less than 15% [15]. Studies have not definitively resolved the uncertainty of which protocol has a higher success or side effect rate in MTX administration [10,16]. The efficacy of MTX therapy is quite high, but lower success rates have been reported in patients with high levels of β-hCG [17]. Increased β-hCG levels while under treatment have been noted as an indicator of treatment failure, and this failure can cause tubal rupture [18]. In addition to β-hCG, other hematologic parameters, such as red cell distribution width, mean platelet volume, and neutrophil-to-lymphocyte ratio, may provide valuable information about the efficacy of MTX therapy [2].

Methotrexate therapy is a treatment that triggers the inflammatory process [19]. Inflammatory markers have been investigated as markers of prognosis in ectopic pregnancy [4]. The neutrophil-to-lymphocyte ratio (NLR) is an inexpensive and practical parameter that can be easily calculated and has many clinical applications. Neutrophils provide the initial host response to pathogens through different mechanisms, such as chemotaxis, phagocytosis, the release of reactive oxygen species (ROS), granular proteins, and the production and release of cytokines [20]. As a component of the innate immune response, the inflammatory response increases neutrophil-mediated death. Therefore, NLR is often characterized by an increase in the number of neutrophils and a decrease in the number of lymphocytes [15]. This easily accessible parameter is being investigated for many diseases, including ectopic pregnancy, preeclampsia, cardiac diseases, and sepsis [15,20,21]. In a study on ectopic pregnancy published in 2017, researchers found that NLR was significantly increased in patients who had surgery [22]. Based on this information, NLR may be an essential indicator in the prediction of rupture, with the thought that surgical treatment will be performed primarily on ruptured ectopic pregnancies. The importance of NLR and blood parameters in treatment choices has also been studied [15,22,23,24]. Predicting rupture practically with a hemogram and parameters as an initial test before starting treatment can be cost effective.

This study evaluated the clinical outcomes of patients with or without rupture who underwent different MTX protocols during EP. Thus, it aimed to compare the efficacy and side effects of varying MTX dose protocols applied in the treatment of ectopic pregnancy and to determine the values of complete blood count parameters, specifically NLR, that may predict the risk of rupture in patients with tubal EP.

## 2. Materials and Methods

This study retrospectively analyzed 128 patients diagnosed and treated for tubal EP in the Health and Sciences University Adana City Hospital between January 2017 and August 2023. After receiving approval from the hospital’s institutional review board, demographic and clinical patient data were obtained from patient files and hospital records.

Ectopic pregnancy was diagnosed by serial transvaginal ultrasonography and serum β-hCG levels. The results of an initial physical examination, laboratory testing, and sonographic evaluation are used for medical or surgical care decisions. Surgery is undertaken in the following circumstances: hemodynamic instability, evidence of intra-abdominal bleeding, or rupture symptoms. Conservative and medical therapy alternatives are considered for women who are clinically stable and have no signs of rupture and bleeding. The patient’s vital signs and symptoms were assessed, followed by initial β-hCG, complete blood count, and liver and kidney function testing. The measurement of ectopic foci was made with sonographic evaluation to assess the maximum size of the ectopic pregnancy (EP). In cases where women exhibited indications of rupture and bleeding, transvaginal ultrasonography was performed, and the pelvis and Douglas pouch were also assessed to determine the existence of hemoperitoneum. Significant amounts of fluid in the abdomen and an accompanying decrease in hemogram values, severe abdominal pain, and detection of Douglas pouch or hematoperitoneum on abdominal puncture were evaluated as an indication of tubal rupture.

Patients who were hospitalized, hemodynamically stable, and had no signs of tubal rupture or bleeding and contraindications to MTX therapy were treated medically, and their data were fully recorded in the health management system included in this study. Patients who did not show extrauterine pregnancy formation via transvaginal ultrasonography and those treated with expectant management or emergency tubal surgery were excluded from the study. Additional exclusion criteria were ultrasonographic evidence of fetal viability in the ectopic sac, heterotopic pregnancy, and chronic inflammatory or autoimmune disease. Patients with lower β-hCG levels or no obvious focus or a focus smaller than 2 cm were treated with a single dose, and patients with β-hCG levels above 5000 mIU/mL and an ectopic area smaller than 3 cm were treated with a double dose. Patients with ectopic areas larger than 3 cm, β-hCG levels higher than 5000 mIU/mL, or located close to the tubal ectopic interstitial area on ultrasonography were treated with multiple doses. Patients were divided into three groups: single dose (Group 1), double dose (Group 2), and multiple doses (Group 3). All patients were also evaluated according to tubal rupture status.

All laboratory tests were analyzed in the same laboratory. Peripheral blood was taken to measure serum β-hCG levels using automated electrochemiluminescence immunoassays.

### Methotrexate Treatment Protocols

Methotrexate was administered at three levels: single dose, double dose, and multiple doses. The administration of a single dose is determined using the equation 50 mg/m^2^, which is derived based on the individual’s body surface area. In the double-dose protocol, MTX was administered intramuscularly with a dose of 50 mg/m^2^ on days 0 and 4. In the multiple-dose protocol, MTX was administered on the 1st, 3rd, 5th, and 7th days, and 1 mg/kg and 0.1 mg/kg leucovorin were administered on the 2nd, 4th, 6th, and 8th days. Serum β-hCG levels were obtained at baseline and on days 1, 3, 5, and 7, until β-hCG declined 15% from the previous value. MTX protocol failure was defined as β-hCG levels that increase or do not decrease, or the need for surgical intervention.

**Figure 1:**
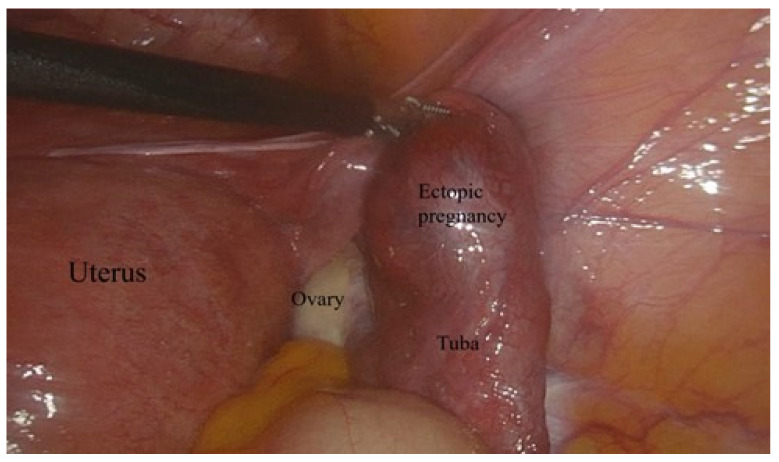
Laparoscopic surgical view of the unruptured tubal ectopic pregnancy.

Laparoscopic surgery was applied to patients who did not respond to treatment or were found to have tubal rupture during follow-up (Figure 1).

The demographic characteristics, ages, parity, body mass indices (BMIs), medical and gynecologic histories (ectopic pregnancy, pelvic inflammatory disease, infertility, intrauterine device, smoking), current sonographic signs (ectopic mass size and site), MTX dosage (mg/day), beta human chorionic gonadotropin (β-hCG) values at diagnosis and during treatment (initial, days 1, 4, and 7), surgical management (salpingectomy or salpingostomy), and laboratory findings (hemoglobin (HGB), hematocrit (HCT), mean corpuscular volume (MPV), platelet count (PLT), white blood cell count (WBC), and neutrophil-to-lymphocyte ratio (NLR) values) were recorded and compared between groups. Additionally, comparisons were made between patients who experienced rupture and those who did not.

This study was conducted in accordance with the Declaration of Helsinki and was reported by the Strengthening the Reporting of Observational Studies in Epidemiology (STROBE). It was approved by the Local Ethics Committee of Health and Science University Adana City Hospital. Patient anonymity and compliance with data protection laws were always maintained. 

Statistical analysis was performed using the SPSS program for Windows, version 22.0 (SPSS Inc., Chicago, IL, USA). For comparison of MTX doses, a one-way ANOVA test was performed for normally distributed data, and Fisher’s least significant difference (LSD) test was utilized as a post hoc test for significant results. The Kruskal–Wallis test was performed for non-normally distributed data, and significant results were evaluated with the Mann–Whitney U test.

In comparing rupture, an independent sample *t*-test was used for normally distributed data, and the Mann–Whitney U test was used for non-normally distributed data. A receiver operating characteristic (ROC) analysis was used to determine the cut-off for NLR value.

## 3. Results

The groups are compared in Table 1 according to age, gravidity, parity, hematologic parameters, and initial β-hCG values. The parity numbers of ectopic pregnancies treated with multiple doses of MTX were higher than of those given a single dose (*p* = 0.047). There was a significant difference between the initial β-hCG values of Groups 1 and 2. The mean baseline β-hCG value of those who received a double dose was 2763.6 ± 3342.46 mIU/mL. The β-hCG values were 6016.1 ± 3244.23 and 8654.1 ± 2304.63 mIU/mL in Groups 2 and 3, respectively. The differences in β-hCG values between groups were statistically significant (*p* < 0.001; Table 1.)

HCT levels were similar between single- and double-dose MTX groups. The HCT levels of Groups 1 and 2 were lower than those of Group 3 (*p* = 0.019). Leukocyte and platelet counts did not differ significantly between groups (*p* > 0.05).

There was a significant difference in the MCV values between the single-dose MTX group and the double- and multiple-dose MTX (*p* = 0.047) groups. The MCV values of the single-dose MTX group were higher than those of the multiple-dose MTX (*p* < 0.05) group.

A comparison of demographic and hematological parameters between patients with and without tubal rupture is shown in Table 2. The mean ages of patients with and without rupture were 31.8 ± 5.3 and 32.0 ± 6.2 years, respectively (*p* = 0.755). The mean hematocrit (HCT) values in patients with and without rupture were 35.5 ± 4.3 and 37.1 ± 3.8, respectively. As a result, the mean hematocrit values of patients without rupture were significantly higher (*p* = 0.038). The NLR distribution of the ruptured group was 3.22 (0.75–11.32), and this ratio was higher than that of the unruptured group at 2.50 (0.30–9.00; *p* = 0.030).

The patients’ gynecological histories and characteristics in managing ectopic pregnancy are compared in Table 3, according to the presence of rupture. There were no significant differences between groups in terms of the number of past ectopic pregnancies, tubal surgery history, intrauterine device (IUD) history, infertility history, smoking, and ectopic pregnancy location (*p* = 0.615, *p* = 0.213, *p* = 0.827, *p* = 0.806, *p* = 0.794, *p* = 0.822, *p* = 0.788, and *p* = 0.763, respectively).

In the non-rupture group, 41 (44.5%) patients were treated with a double-dose protocol, 27 (26%) were treated with multiple doses, and 24 (26.0%) followed a single-dose protocol. In the rupture group, 72.4% (*n* = 26) of patients in Group 1 received a single dose of methotrexate. According to the methotrexate protocol, there was a significant difference between groups (*p* = 0.002). Salpingectomy was not performed in 41 patients (66.1%) without rupture, whereas it was used in 35 patients (97.2%) with rupture (*p* = 0.001). In this group, only one patient underwent salpingostomy. In the rupture group, 5 (13.8%) patients underwent dilatation and curettage (D/C) for diagnosis, compared to 27 (86.10%) patients in the non-ruptured group (*p* = 0.015).

**Figure 2:**
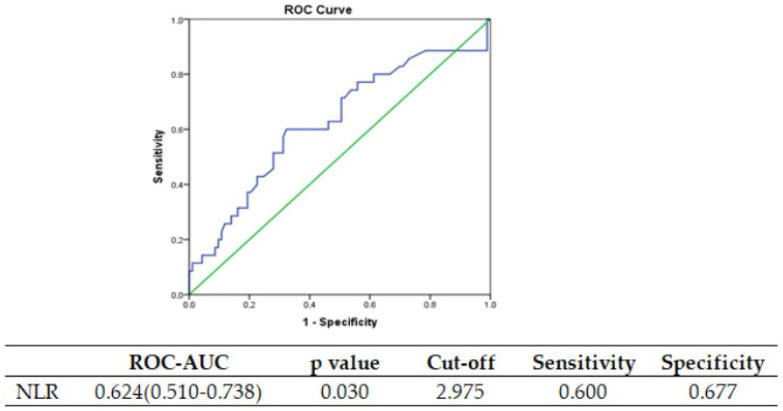
The area under the receiver operating characteristic curve and cut-off values of NLR for tubal rupture.

NLR exhibited a noteworthy increase in the ruptured group. When considering the presence of rupture, the diagnostic performance of the NLR variable yielded a value of 0.624 (0.771–0.956) in terms of ROC-AUC, and an optimal cut-off value of 2.974 was identified (refer to Figure 2 for the ROC curve).

## 4. Discussion

Treatment modalities for EP may range from standby therapy to radical surgery, depending on the clinical condition of the patients [25]. In clinical practice, β-hCG is the primary biomarker employed, and basal β-HCG levels are predictive of a reliable treatment with MTX [26]. It has been reported that treatment success rates with MTX depend on factors such as the initial β-hCG concentration, the size of the extrauterine mass, and previous ectopic pregnancy history [3]. Although Song et al. stated that a double-dose protocol was more successful when β-hCG levels were above 5000 mIU/L, a comprehensive meta-analysis showed no difference between treatment protocols [17,27]. Again, this meta-analysis stated that a double dose should be preferred if β-hCG levels are between 3600 and 5000 mIU/L [17]. In our study, the highest β-hCG level was in the multiple-dose group, and the lowest β-hCG level was in the single-dose group (*p* < 0.001).

The most critical parameter for the choice and success of treatment of an ectopic pregnancy is the presence of rupture [25]. As for treatment failure, the presence of a rupture requiring surgery has been noted in many studies [11,17,22]. An increasing number of unruptured ectopic pregnancies are seeking MTX treatment, but it is unclear which patients will benefit most. Chen et al. compared the baseline demographics and β-hCG trends of patients with ruptured EPs after MTX treatment with patients whose EPs recovered and found that the β-hCG change was less pronounced in the ruptured cases [28]. Although many studies have reported a positive correlation between β-hCG values and a risk of rupture, a definite threshold for the risk of rupture cannot be determined. A study conducted in the United States reported that β-hCG levels could not predict the risk of rupture in EP [18]. Our research showed no statistically significant difference in β-hCG levels in patients with and without rupture before, after, and during the follow-up periods.

A study published in 2020 reported that the mean age of women diagnosed with EP was 25.29 years [14]. In our study, the mean age was 31.9 years. When the mean age was compared according to MTX dose, it was found that the mean age was higher in patients who received multiple doses compared to patients who received single or double doses of MTX. However, this difference was not statistically significant. Yang et al. stated that ectopic pregnancy rupture was correlated with parity and advanced maternal age. In the current study, parity was significantly higher in patients who received multiple MTX doses than in the other groups (*p* = 0.048) but not found to be correlated with rupture.

Since tubal rupture prediction is essential, studies have focused on this issue. Complete blood count parameters were investigated as an inexpensive and easy predictor marker. A study comparing hematological parameters in patients diagnosed with EP who underwent surgical methods and MTX treatments reported that MPV, RDW, and NLR values were significantly lower in patients who underwent surgical procedures than those who underwent MTX treatment [4]. Another study showed that platelet, neutrophil, and NLR levels were significantly higher in EP patients with rupture than in patients without rupture [23]. Kan et al. reported that NLR and PLR were positively correlated with serum β-hCG levels and tubal diameter measurement in ruptured tubal pregnancies [23]. In another study, the authors reported that high RDW and MPV values in stable tubal EPs were independently associated with the surgical versus methotrexate treatment options; also, NLR levels were significantly increased in the methotrexate group, but PLR levels were not significantly different [4]. Kanmaz et al. explained the elevated neutrophil-to-lymphocyte ratio (NLR), reported to be elevated in patients requiring surgical treatment, by the prolonged inflammatory response. Consistent with all these data, in our study, all parameters were also compared according to the presence of rupture. HCT was significantly lower and NLR was significantly higher in patients with rupture (*p* = 0.038 and *p* = 0.030, respectively).

The strengths of this study include the evaluation of patients according to treatment modalities and rupture status, without a lack of data on patient groups, including all three methotrexate protocols. Therefore, this study showed that neutrophil-to-lymphocyte ratio (NLR) assessment can serve as a significant indicator in patients with rupture, irrespective of the treatment dosage.

The present study is subject to some limitations, including its retrospective design and the fact that it was conducted at a single center. There is a requirement for multicenter prospective research to ascertain criteria that might effectively predict the occurrence of tubal ectopic pregnancy rupture. Nevertheless, a significant advantage of our clinic is being situated within the main center where patients receive ongoing care and in a hospital that provides a large geographical area.

## 5. Conclusions

In the current study, we found that NLR could indicate the risk of rupture. However, there was no consistent relationship between MTX treatment dose and β-hCG. Although the relationship between β-hCG and EP rupture was mostly analyzed, we think that NRL is an important parameter to show this relationship.

## Figures and Tables

**Table 1:** Comparison of the findings according to MTX administration in EP.

	Total	Group 1 Single-Dose MTX (*n* = 50), Mean ± SD	Group 2 Double-Dose MTX (*n* = 48), Mean ± SD	Group 3 Multiple-Dose MTX (*n* = 30), Mean ± SD	*p*-Value
Age	31.91 ± 5.93	31.06 ± 6.14	32.29 ± 6.19	33.04 ± 5.09	0.340
Gravida	2.55 ± 1.53	2.33 ± 1.56	2.39 ± 1.71	3.14 ± 1.04	0.114
Parity	1.11 ± 1.053	0.90 ± 0.968	1.03 ± 1.158	1.57 ± 0.945	0.048
Initial β-hCG value (mIU/mL)	5060.9 ± 1246.70	2763.6 ± 3342.46	6016.1 ± 3244.23	8654.1 ± 2304.63	<0.001
HGB (g/dL)	12.1 ± 1.47	11.8 ± 1.62708	12.4 ± 1.32	12.4 ± 1.28	0.076
HCT (%)	36.7 ± 4.05	37.3 ± 3.27	37.7 ± 3.45	35.5 ± 4.68388	0.019
MCV (fL)	83.54 ± 9.71	84.92 ± 5.95	82.99 ± 10.98	79.9 ± 12.2	0.047
MCH (pg)	27.92 ± 3.27	28.2280 ± 2.41289	28.03 ± 4.03	27.33 ± 3.17	0.505
MPV (fL)	9.84 ± 1.27	9.6378 ± 1.21803	9.92 ± 1.29	9.99 ± 1.36	0.413
PLT (K/mL)	273 ± 624	262 ± 647	274 ± 548	284 ± 617	0.278
WBC (K/μL)	11.235 ± 11.059	12.594 ± 12.451	10.928 ± 12.691	9.672 ± 2.386	0.111
PDW	14.13 ± 4.54	13.91 ± 5.06	14.6 ± 5.02394	13.78 ± 2.6	0.529
NLR	3.57 ± 1.63	3.67 ± 1.98	2.8 ± 1.72	3.28 ± 1.88	0.192

*n*: number of patients in the group; mean ± SD: mean ± standard deviation; β-hCG: beta human chorionic gonadotropin; HGB: hemoglobin; HTC: hematocrit; MCV: mean corpuscular volume; MCH; mean corpuscular hemoglobin; MPV: mean platelet volume; PLT: platelet; WBC: white blood cell count; PDW: platelet distribution width; and NLR: neutrophil-to-lymphocyte ratio.

**Table 2:** Comparison of demographic and hematological parameters between patients with and without tubal rupture.

Variables	Rupture		*p*-Value
	No *n* = 92 (Mean ± SD)/(Min − Max)	Yes *n* = 36 (mean ± SD)/(Min − Max)	
Age	32.03 ± 6.21	31.85 ± 5.35	0.755
BMI	25.9 ± 3.28	24.1 ± 2.49	0.092
Parity	1.00 (0.00–5.00)	1.00 (0.00–3.00)	0.533
HCT (%)	37.188 ± 3.845	35.529 ± 4.373	0.038
HGB (g/dL)	12.298 ± 1.407	11.909 ± 1.600	0.182
MCV (fL)	86.50 (27.70–96.90)	84.40 (73.50–97.30)	0.340
MCH (pg)	29.00 (10.50–32.90)	28.20 (23.10–33.10)	0.246
PLT (K/mL)	266 (150–443)	273 (160–476)	0.716
WBC (10^/uL)	8.870 (7.31–94,600)	9.900 (5690–46,800)	0.057
MPV (fL)	10.10 (6.80–12.80)	9.80 (7.20–12.60)	0.263
PDW	12.90 (8.80–44.30)	13.50 (9.00–41.30)	0.567
NLR	2.50 (0.30–9.00)	3.22 (0.75–11.32)	0.030
Initial β-hCG value (mIU/mL)	5.171 (90–11.991)	4.296 (33–20.421)	0.879
Time to negative β-hCG mIU/mL	36 (10–155)	32 (13–114)	0.303
1 day	3.558 (110–11.991)	1.921 (560–19.097)	0.669
4 days	5.225 (102–15.502)	4.996 (658–21.956)	0.915
7 days	5.856 (33–13.854)	3.649 (102–14.264)	0.428
9 days	7.510 (76–48.604)	3.178 (145–11.222)	0.233

*n*, %: number of patients in the group and percentage; BMI: body mass index; HTC: hematocrit; HGB: hemoglobin; MCV: mean corpuscular volume; MCH; mean corpuscular hemoglobin; PLT: platelet; WBC: white blood cell count; MPV: mean platelet volume; PDW: platelet distribution width; NLR: neutrophil-to-lymphocyte ratio; and β-hCG: beta human chorionic gonadotropin.

**Table 3:** Comparison of the medical histories and ectopic pregnancy treatments of patients with and without rupture.

Variables		Rupture		*p*-Value
		No (*n* = 92) *n* (%)	Yes (*n* = 36) *n* (%)	
History of ectopic pregnancy	1	89 (96.7)	33 (94.3)	0.615
	≥2	3 (3.3)	3 (5.7)	
History of tubal surgery	No	89 (95.7)	31 (88.6)	0.213
	Yes	3 (3.3)	5 (11.4)	
History of IUD	No	75 (80.6)	28 (80.0)	>0.999
	Yes	17 (19.4)	8 (20.0)	
History of infertility	No	72 (78.5)	30 (82.9)	0.806
	Yes	20 (21.5)	6 (17.1)	
History of PID	No	76 (82.8)	31 (85.7)	0.794
	Yes	16 (17.2)	5 (14.3)	
Smoking	No	69 (74.2)	27 (77.1)	0.822
	Yes	23 (25.8)	9 (22.9)	
Tubal lumen measurement *	Unmeasurable	42 (45.6)	13 (36.2)	0.788
	<35 mm	35 (38.0)	15 (41.6)	
	≥36 mm	15 (16.3.)	8 (22.2)	
Ectopic site	Right tuba	45 (48.9)	17 (48.6)	>0.999
	Left tuba	47 (51.1)	19 (51.4)	
Methotrexate regimen	Single dose	24 (26.0)	26 (72.4)	0.002
	Double dose	41 (44.5)	7 (19.4)	
	Multiple doses	27 (29.3)	3 (8.3)	
Salpingectomy	No	71 (77.2)	1 (2.9)	<0.001
	Yes	21 (22.8)	35 (97.2)	
D/C	No	53 (66.2)	31 (86.10)	0.015
	Yes	29 (33.8)	5 (13.8)	

* Measurement with transvaginal ultrasonography. (*n*, %): number of patients in the group and percentage; IUD: intrauterine device; PID: pelvic inflammatory disease; and D/C: dilatation and curettage.

## Data Availability

The data supporting this study’s findings are available from the corresponding author, [S.S.], upon reasonable request.
